# Practical Application of Electrolysis

**Published:** 1888-07

**Authors:** W. B. Ames

**Affiliations:** Chicago, Ill.


### The Practical Application of Electrolysis.

BY W. B. AMES, D.D.S., CHICAGO, ILL.

[Read before the Michigan State Dental Association at Ann Arbor, Mich., March 20, 1888.]

There are two important uses. As applied to the bleaching of discolored teeth, by getting nascent oxygen in contact with the surface to be bleached. To accomplish this, after filling the root flood the cavity with acidulated water (one drop to the ounce, to render it a better electrolyte) then place a metal electrode connected with the negative pole of the battery in contact with the moisture somewhere near the margin of the cavity and pass a platinum needle, connected with the positive pole of the battery, over the surface to be bleached. Upon closing the circuit the oxygen of the water is liberated at the positive pole near the surface to be bleached, and the hydrogen is liberated at the negative electrode outside the cavity. In all processes of bleaching it is the nascent oxygen on which we depend for our results.

In the treatment of pyogenic surfaces, the object is to liberate in contact with the tissues some potent agent such as nascent oxygen, ozone or iodine.

In the treatment of pyorrhœa we can electrolytically decompose the contents of the pockets about the roots of the teeth by passing into these pockets a platinum electrode connected with the positive pole of the battery, passing a current from it to a sponge electrode connected with the negative pole and held by the patient in the palm of the hand or against the face, thus securing nascent oxygen and ozone in the pocket about the needle. An increased stimulation can be obtained by decomposing in the same manner a solution of iodide of potassium placed in the pocket. This can be conveniently done by picking up a few crystals of the iodide upon the moistened end of the positive electrode and carrying it to the pocket, its fluid contents forming a solution. The aqueous solution of iodine obtained by this electrolytic decomposition is an ideal aqueous solution and is much superior to the alcoholic solution.

The apparatus necessary for this is the flexible platinum needle to be connected with the positive pole of the battery, and a sponge electrode to be connected with the negative pole, to be used as stated. The sponge electrode should be moistened with a warm solution of common salt in water. This decreases the resistance, thus allowing of a milder current than could otherwise be used. Two fresh cells will usually accomplish the result, but if not fresh three or more will be needed. The principle upon which this process depends is the law governing electrolytic decomposition; the positive elements of an electrolyte appear at the negative pole and the negative elements at the positive pole in the decomposition of any fluid conductor.

For the treatment of pus sacks at the apical end of roots having an outlet through a fistula, I would use an olive pointed platinum electrode of a size that could be readily passed through the fistula, decomposing the pus and other contents, or a solution of iodide of potassium placed there in the same manner as in the case of pyorrhœa pockets.

DISCUSSION.

*Dr. J. Taft:* There are cases in which this method seems to be most invaluable. We must, however, remember that it is not of general application, for we are liable to attempt impractical uses of it. How far will the application be liable to reach? Will it go to the bottom of the pocket? If not, how shall we accomplish that object? We might wrap the electrode in silk so as to insulate all but the very end. Then the current can be placed at any point desired. In breaking up tumors this process may be used. I have had no experience with malignant tumors, have seen several cases of benign tumors broken down and healthy tissues spring up in their places. Think it in many cases better than to cut them out. Again, in the case of diseased gums you may decompose the contents of the enlarged parts of the gums. The question arises, how much can be gained by decomposition over some other method? If you get simply free oxygen you have nothing better than can be done by peroxide of hydrogen. We destroy the matter which may be there. It is the persistent presence of the irritant that causes the trouble. Get the evil out and the tissues can rally. Am not sure but that the decomposition of iodine would accomplish too much. It is liable to do more than is necessary. The nascent iodine would be more escharotic in its influence than is needed. It is very powerful. The cases require special thought and care. We should apply remedies with caution. There is often more danger of an excess of treatment than of a falling short.

*Dr. W. B. Ames:* A needle may be used which is insulated except at the extreme point. A nice manner of getting this insulation is to use a steel or iron probe which has been gold plated for about an eighth of an inch at its extreme end. In use, the unplated part of this brooch will very soon oxidize, the oxide forming a very good insulation, leaving the plated extreme to do the entire work. For some cases I only decompose the contents of the pyorrhœa pockets and pus sacks, but in cases of long standing I decompose a solution of iodide of potassium placed there, aiming to get the stimulating effects of the nascent iodine. The aqueous solution of iodine obtained by the electrolytic decomposition of the solution of iodide of potassium has the stimulating properties without having the same escharotic effects as an alcoholic solution of iodine. Have never seen any bad results from this use of iodine. Theoretically it would seem that the decomposition would not be the same upon all surfaces of the needle immersed into the contents of the pocket, but it is so nearly so, practically, that a needle partially insulated is not needed for this purpose. There is undoubtedly a stimulation from the passage of the galvanic current, but we do not know to what extent this enters as a factor in producing the results. In the disinfection of root canals by the electrolytic decomposition of the putrid mass contained in them a partially insulated needle, such as described, is useful for the reason that in this case it is desirable to bring about the decomposition most intensely at the extreme end of the needle. By passing this gradually to the apex the contents are thoroughly decomposed or at least disinfected, and at its thorough removal the root canal can usually be immediately filled. This applies, of course, only to cases where the root canal can be easily traversed by the needle. Have found it of quite extensive use in my practice.

